# Barriers and facilitators to return to work in young and middle-aged patients after PCI: a qualitative study based on the COM-B model

**DOI:** 10.1038/s41598-026-51049-1

**Published:** 2026-04-29

**Authors:** Lanfang Zhang, Yue Liu, Wenting Zhang, Yi Cui, Yinling Zhang, Xiaolan Guo

**Affiliations:** 1 Department of Nursing, Air Force Medical University, Xi’an, China; 2 Department of Cardiovascular, the Second Affiliated Hospital of Air Force Medical University, Xi’an, China

**Keywords:** Return to work, Coronary heart disease, PCI, COM-B model, Qualitative research, Cardiology, Health care, Psychology, Psychology

## Abstract

The issue of younger onset coronary heart disease (CHD) is becoming increasingly prominent. Percutaneous coronary intervention (PCI) has proven to be an effective treatment for CHD, significantly reducing mortality risk. Young and middle-aged patients with CHD typically need to return to work (RTW) following PCI; however, the proportion of those who achieve a timely return remains low. Further research is needed to identify factors influencing RTW decisions post-PCI. This study adopted a qualitative research design to investigate the barriers and facilitators influencing the RTW among young and middle-aged patients with CHD following PCI. 19 patients had undergone PCI were purposively selected from a tertiary hospital in Xi’an, China, and semi-structured interviews were conducted with them. The data were analyzed using an inductive thematic approach. Thirteen themes were identified and directly mapped onto the COM-B model (Capability, Opportunity, Motivation-Behavior). The constructs of the COM-B model revealed three primary barriers: physical limitations, insufficient knowledge, and lack of employment opportunities. Key facilitators for RTW included health-oriented behavioral modifications, manageable workloads, strong social support networks, family and professional responsibilities, optimism regarding RTW prospects, belief in the positive outcomes associated with RTW, the objective of securing economic income, and habitual behaviors influenced by professional identity. Additionally, two factors served dual roles as both facilitators and barriers: descriptive norms and emotional responses. This study identified key barriers and facilitators influencing RTW among young and middle-aged patients after PCI. The findings indicate that RTW behavior in this population is shaped by the interplay of capability, opportunity, and motivation. Healthcare providers should focus on enhancing patients’ cardiac function, delivering relevant work-related knowledge and health education, and strengthening social support systems to improve both their capability and opportunity for returning to work. Furthermore, promoting positive beliefs and attitudes regarding RTW while alleviating negative emotions can further bolster their motivation to re-enter the workforce.

## Introduction

Coronary heart disease (CHD) is characterized by high incidence and mortality rates, making it one of the leading causes of premature death in populations worldwide^1,2^. Currently, approximately 197 million individuals globally are living with CHD^3^. In China, CHD presents a significant public health challenge. The prevalence of CHD among Chinese residents aged 18 and older stands at 758 per 100,000, equating to roughly 11.39 million patients^4^. Notably, there is an increasing trend towards younger age groups being affected by this condition. Research indicates that the incidence of CHD among individuals under the age of 55 has been rising annually^5^. Specifically, the average annual growth rate for CHD incidence in residents aged 20–49 years reaches as high as 6.37%^6^. Consistent with global trends, all metrics—including prevalence, incidence, mortality rates, and associated economic costs—are on the rise within the young and middle-aged population (ages 18–60)^7,8^. Percutaneous coronary intervention (PCI), recognized as a primary treatment modality for coronary heart disease^9^, has been shown to significantly alleviate angina symptoms while reducing patients’ risk of mortality^10,11^. In China alone, approximately 1.421 million patients undergo coronary interventions each year^4^.

Young and middle-aged individuals bear considerable family and social responsibilities, positioning themselves as the primary contributors to the creation of social wealth and serving as a vital pillar for both familial economic stability and emotional support^12^. Following PCI, these individuals often face the challenge of reintegrating into society and resuming work while managing their health condition^1^. Return to work (RTW) refers to patients returning to their original jobs or taking up new paid positions^13^. This process holds significant importance in enhancing economic benefits, improving quality of life, alleviating negative emotions, and fulfilling personal self-worth^14,15^.

Currently, the proportion of young and middle-aged patients with CHD who return to work within a specified timeframe is relatively low. A meta-analysis showed that the prevalence of RTW among patients with CHD is 58%^16^. Furthermore, a study involving 3291 patients from 24 European countries revealed that 76% of these individuals returned to work between six months and three years post-discharge^17^. Additionally, a prospective cohort study revealed that approximately 89% of patients resumed work within three years following PCI^18^. Among Iranian myocardial infarction patients, it was found that 77% returned to work after one year^19^. Research conducted by Chinese scholars has shown that only about 60% of young and middle-aged CHD patients were able to return to work three months after discharge^13,20^, while the RTW rate for those undergoing PCI was merely 55.9% one year post-discharge^21^.

From a clinical rehabilitation perspective, RTW serves as a crucial indicator of patients’ social-functional recovery^22,23^. The observed low RTW rate may reflect shortcomings in current postoperative rehabilitation programs designed to restore vocational capacity. This underscores the urgent need to optimize intervention strategies through a comprehensive analysis of contributing factors. Furthermore, considering the pivotal role played by young and middle-aged adults in social productivity, their lower RTW rates not only exacerbate familial medical burdens but also entail potential losses of labor resources at the societal level. Identifying the key determinants can provide a theoretical foundation for developing targeted social support policies.

Presently, research on the factors influencing RTW in patients following PCI primarily relies on cross-sectional surveys^17,18,20,24^. These studies examine a range of factors, including sociodemographic variables, disease-related characteristics, psychosocial factors, and occupational aspects^20,24–27^. A notable limitation of previous investigations is the lack of qualitative research. Quantitative studies predominantly focus on identifying influencing factors; consequently, they often fail to capture patients’ subjective experiences regarding RTW, as well as the complex considerations involved in their decision-making processes and the dynamic interactions among various factors. For middle-aged and younger patients, RTW is not solely dependent on physical recovery; it also encompasses career aspirations, familial responsibilities, and societal values—dimensions that are poorly reflected in quantitative data. Qualitative research methods, such as in-depth interviews, can reveal these individuals’ genuine dilemmas, internal conflicts, and needs related to balancing rehabilitation with RTW. This approach addresses the shortcomings of quantitative research in explaining “how” and “why,” thereby providing a foundation for developing targeted RTW intervention programs aimed at effectively facilitating vocational rehabilitation for this population.

**Fig. 1:**
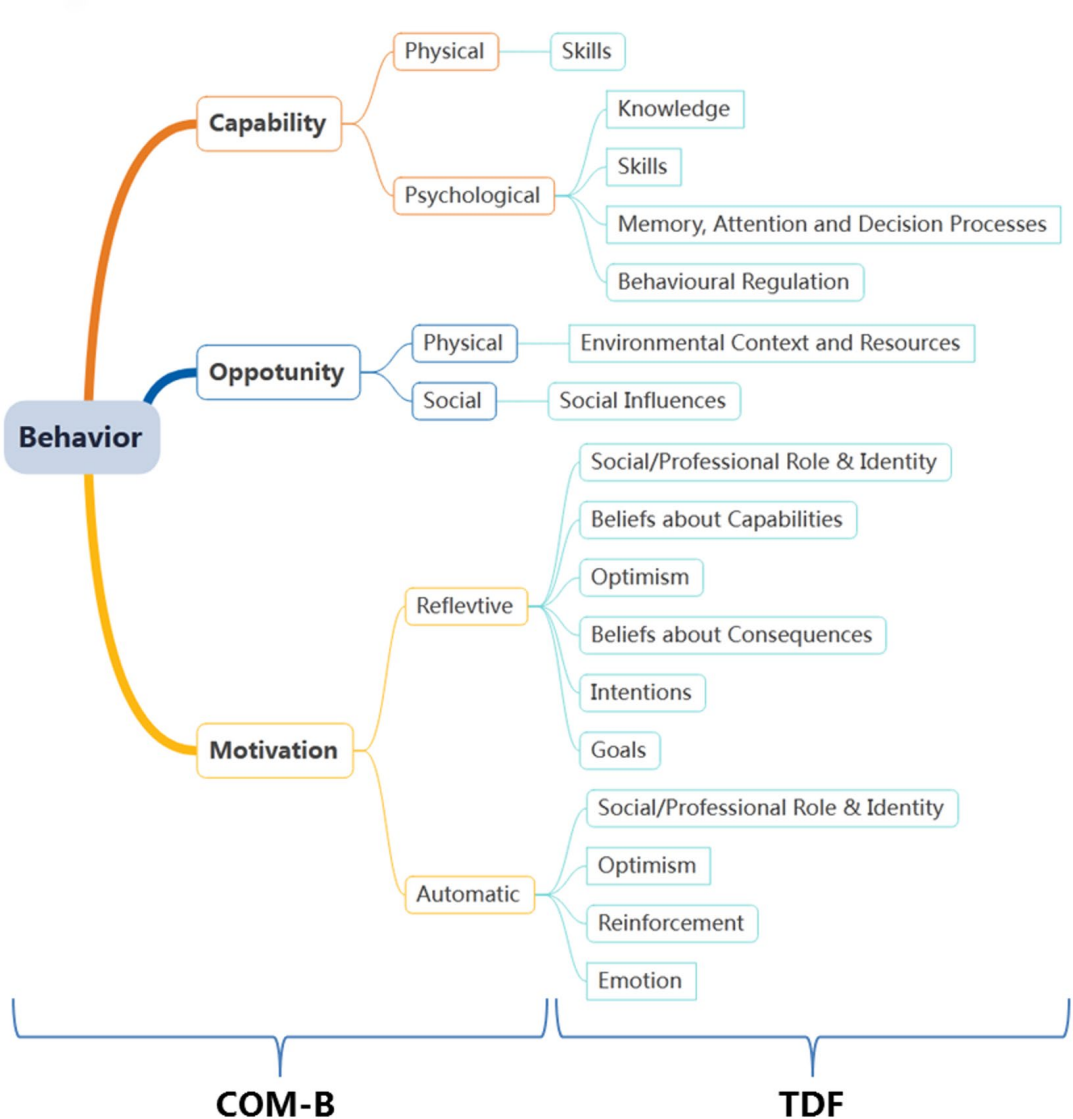
Map of TDF to COM-B model.

Evidence indicates that applying theoretical frameworks to identify the mechanisms underlying behavioral change enhances our understanding of the behavioral change process and helps determine its influencing factors. This, in turn, facilitates the development and formulation of targeted intervention strategies^28,29^. The Behavior Change Wheel (BCW) advocates for utilizing the Capability, Opportunity, Motivation-Behavior (COM-B) model to elucidate the influencing factors that can serve as targets for behavioral change interventions. This model underscores that behavior determinants encompass individuals’ physical and psychological capabilities, as well as their physical and social opportunities, alongside reflective and automatic motivations^30^. The Theoretical Domains Framework (TDF) represents a significant comprehensive framework within behavioral change theories and constitutes a core component of BCW theory^31^. For further details, refer to Fig. 1. Currently, both COM-B and TDF are extensively employed in managing cardiovascular disease^32,33^. They not only analyze individual behavior-influencing factors but also guide the design of intervention programs^34–37^. However, studies investigating RTW behavior’s influencing factors through the lens of BCW theory remain limited.

Consequently, this study aims to explore in depth the barriers and facilitators to returning to work among young and middle-aged patients with CHD after PCI, through qualitative interviews grounded in the TDF. This work will establish a foundation for developing precise and effective behavior change interventions.

## Methods

### Design

This study was grounded in the constructivist paradigm. A descriptive qualitative research design was employed to thoroughly investigate the factors that facilitate or impede patients’ RTW following interventional surgery. Theoretically, the COM‑B model served as the overarching theoretical framework for understanding this behavior. Meanwhile, the TDF guided both data collection and analysis throughout the study. To ensure transparency in reporting, this research adhered to the Consolidated Criteria for Reporting Qualitative Research (COREQ) guidelines^38^.

### Participants and recruitment

Participants were recruited from the Department of Cardiovascular Medicine of a university-affiliated tertiary hospital in western China from January to March 2025. To ensure maximum variation sampling, purposive sampling was employed to select patients representing diverse characteristics (including age, education level, and occupation). The inclusion criteria were: (a) a confirmed diagnosis of coronary heart disease who had undergone PCI, (b) aged 18–59 years (this age range was selected to focus on the core working-age population in China, where the statutory retirement age at the time of the study was typically 60 for men, 55 for female cadres, and 50 for female workers), (c) employed prior to the procedure (including self-employed individuals and migrant workers), and (d) adequate comprehension and communication skills. Exclusion criteria comprised: (a) diagnosed mental illness or cognitive impairment, (b) significant comorbidities such as malignant tumors, respiratory failure, or severe hepatic/renal insufficiency, (c) physical disability, and (d) anticipated employment contract expiration or retirement within six months post-discharge.

The Readiness for Return-to-Work (RRTW) model conceptualizes return to work as a dynamic process starting with a precontemplation stage, during which individuals have not yet considered resuming work^39^. Accordingly, to obtain comprehensive experiential data, interviews were conducted at varying time points post-PCI, ranging from 1 to 122 days after the procedure. This included both inpatients during their hospital stay and participants who had been discharged and were in the early recovery phase at home. This approach enabled us to capture both immediate post-procedure contemplations and the evolving early experiences related to the return-to-work process. The specific interval since PCI for each participant is presented in Table 1.

### Data collection

Nurses screened patients who met inclusion criteria (a) and (b) from the medical record system of the Department of Cardiovascular Medicine. The first author subsequently contacted these patients to confirm their eligibility for criteria (c) and (d), inquired about their availability for interviews, and conducted interviews via WeChat video. Patients selected for face-to-face interviews were recruited from inpatients within the cardiovascular department. Nurses verified their eligibility in person, while the first author conducted face-to-face interviews at times that did not conflict with their treatment schedules. For all eligible patients, provided an introduction to the study, obtained informed consent, and recorded interviews.

The interview guide was designed based on the COM-B model and the TDF (Appendix 1). The interview questions were refined to eliminate redundancy and consolidated into eight coherent questions that are more comprehensible for patients:

1. *Do you plan to return to work? When do anticipate resuming your duties? How believe it is that you will return to work? What circumstances have previously hindered your ability to return or caused interruptions in your employment?*
2. *How do you feel about the prospect of returning to work? Do you have any concerns regarding this transition? Will these feelings or emotions affect your willingness to return to work?*
3. *Do you think returning to work is important? Why do you think so? Do you believe that this decision necessitates careful consideration and a weighing of its advantages and disadvantages? (Tips: Is making this decision challenging for you? ) What decisions must be addressed during the process of returning to work?*
4. *What benefits do you foresee from returning to work? What consequences might arise if you choose not to return? How could this impact your health overall? (Tips: Consider both the potential benefits and drawbacks.)*
5. * Which factors do you believe will motivate your return to work*,* and which may impede it? Do you feel that you possess adequate decision-making authority and resources concerning your return-to-work plans? What specific resources are included in this assessment? How confident are you in managing any difficulties or challenges that may emerge upon re-entering the workforce?*
6. *Do your family members*,* friends*,* colleagues*,* and healthcare providers support your decision to return to work? Why? What type of care and support has your workplace or employer provided you with during this transition? Are you aware of anyone who has been diagnosed with coronary heart disease or who has undergone surgery for other medical conditions? Are they currently employed? Have there been any repercussions associated with not returning to work? Conversely*,* have those who have returned managed to recover physically and perform effectively in their roles? Will you consider comparing yourself to them when determining the appropriate time for your own return to work?*
7. *In light of your physical condition following surgery*,* do you believe that adjustments are necessary regarding your job responsibilities? What specific modifications do you think are required? Which skills or capabilities do you feel need enhancement in order to fulfill the demands of returning to work successfully? How do you intend to balance professional obligations with health considerations?*
8. *What measures or strategies do you propose could facilitate the reintegration of patients into the workforce after undergoing interventional surgery?*

The questioning sequence for each participant was tailored based on their responses. The duration of the interviews ranged from 15 to 49 min. All interviews were conducted by the first author to ensure consistency in data collection.

#### Data analysis

Data analysis was divided into two phases: (1) Braun & Clarke’s^40^ inductive thematic analysis, and (2) deductive mapping to the TDF and COM-B Model.

Phase 1: Inductive Thematic Analysis (a) Familiarization with the Data: Within 24 h of each interview, the first author transcribed the recordings verbatim, and the second author verified the accuracy of the transcribed text. The research team (first and second authors) then immersed themselves in the data through repeated reading of the transcripts. (b) Generating Initial Codes: The first author systematically coded the full dataset line by line using NVivo 12.0, generating initial codes that captured meaningful features. (c) Searching for Themes: The first author collated related codes into candidate themes. The entire research team then reviewed and discussed these candidate themes to identify coherent overarching themes. (d) Reviewing Themes: The research team collaboratively reviewed candidate themes against the coded data extracts and the full dataset to ensure they formed a coherent pattern and accurately represented the data. (e) Defining and Naming Themes: Clear definitions and labels for each final theme were refined through team discussion to capture their core essence. (f) Producing the Report: Final themes, supported by illustrative participant quotes, were synthesized and formed the basis of the Results section.

Phase 2: Deductive Mapping to the TDF and COM-B Model The themes derived from Phase 1 were treated as subthemes and deductively mapped onto the corresponding domains of the TDF by two researchers working independently, based on the definitions of each TDF domain. These subthemes were then further categorized into the three core components of the COM-B model (Capability, Opportunity, and Motivation) according to the established theoretical links between TDF domains and the COM-B model, with these three components serving as the main overarching themes in this study. Any discrepancies during the mapping and categorization processes were resolved through team discussion until consensus was achieved.

Data collection and analysis proceeded concurrently. The sample size was determined by data saturation: specifically, after analyzing at least ten interviews, data collection ceased when no new codes or themes emerged in three consecutive interviews^41^.

### Ethical considerations

This study received ethical approval from the Tangdu Hospital, Fourth Military Medical University (K202503-47). All methods were performed in accordance with the relevant guidelines and regulations. Prior to the interview, all participants provided informed consent. They were made aware that they could withdraw from the study at any time or decline to answer any questions. Any personal information disclosed during the interview was removed, and participant transcripts were identified by research numbers rather than names to ensure patient privacy protection. Access to the data was restricted solely to research team members, and all data related to this study were securely stored on password-protected computers.

### Methodological rigor

Recognizing the researcher as the primary instrument in qualitative inquiry, we actively engaged in reflexivity to ensure the trustworthiness of the study. The first author maintained a reflective journal throughout the study to sustain a reflexive stance and explore participants’ experiences from their own perspective. The research team, with backgrounds in nursing and qualitative methodology, regularly reviewed interview transcripts, codes, and emerging themes to challenge individual interpretations and mitigate potential bias.

This study implemented quality control across four key aspects: credibility, dependability, confirmability, and transferability, in accordance with established standards for qualitative research^42^. In terms of credibility, the first author participated in multiple training sessions related to qualitative research and possesses nursing experience in the cardiology department, thereby demonstrating competence in conducting qualitative studies. Furthermore, the first author had no professional intersections or involvement in patients’ clinical treatment, which alleviated any concerns interviewees might have had during the interviews and enhanced the credibility of their responses. To ensure dependability, the reflexive practices described above were employed, and the interview content was meticulously reviewed. NVivo12.0 was utilized for data management to facilitate traceability. This study provided illustrative quotes and conducted repeated verifications of statements with interviewees to enhance confirmability. Regarding transferability, researchers offered detailed descriptions of the interview outline as well as data collection and analysis processes. This thorough documentation enables other researchers to assess whether essential findings are applicable to similar contexts and populations.

## Results

### Participants

A total of twenty eligible patients were invited to participate in this study. One patient declined the invitation due to physical discomfort. Consequently, a total of nineteen patients were interviewed, aged between 35 and 58 years, with a mean age of 49 years, and only two participants were female. Eighteen patients resided in urban areas, and 63.16% reported a monthly income ranging from 5,000 to 10,000 yuan. Two interviews were conducted face-to-face during hospitalization, while the remaining seventeen patients participated via video conferencing. The majority (76.47%) of the discharged patients had returned to work, with an average postoperative return-to-work time of 14.92 days (see Table 1).

**Table 1:** Demographic characteristics of the participants (*N* = 19).

No	Gender	Age	The number of stents	Job before PCI	Working years	Monthly income (CNY)	Educational level	Residence	Interview formats	Days post-PCI	Postoperative RTW time
P1	Male	58	3	Teacher	39	5,000–10,000	Undergraduate degree	Urban area	Video	43	1month
P2	Male	53	1	driver	33	5,000–10,000	High school diploma	Urban area	Video	43	Not returned to work
P3	Male	51	2	teacher	33	5,000–10,000	College diploma	Urban area	Video	56	1month
P4	Male	48	1	Civil servant	29	5,000–10,000	Undergraduate degree	Urban area	Video	67	2days
P5	Male	51	2	Power plant worker	35	5,000–10,000	College diploma	Urban area	Video	81	7days
P6	Male	47	1	Transportation engineering professional	25	10,000–15,000	College diploma	Urban area	Face-to-face	1	/
P7	Male	56	2	Civil servant	34	5,000–10,000	Undergraduate degree	Urban area	Video	119	1month
P8	Male	41	1	Highway administrator	16	5,000–10,000	High school diploma	Urban area	Video	122	16days
P9	Male	57	1	Bridge worker	5	5,000–10,000	Junior high school diploma	Rural area	Face-to-face	2	/
P10	Male	35	2	Engineering Administrator	10	10,000–15,000	Undergraduate degree	Urban area	Video	106	20 days
P11	Male	50	1	Civil servant	30	5,000–10,000	High school diploma	Urban area	Video	109	7days
P12	Male	46	1	Supermarket owner	3	>20,000	Junior high school diploma	Urban area	Video	18	7days
P13	Male	51	2	Bank staff	29	10,000–15,000	Undergraduate degree	Urban area	Video	43	8days
P14	Female	51	1	teacher	31	5,000–10,000	Undergraduate degree	Urban area	Video	78	Not returned to work
P15	Male	58	4	Civil servant	35	5,000–10,000	Undergraduate degree	Urban area	Video	85	Not returned to work
P16	Female	43	1	Cashier	21	<5000	Undergraduate degree	Urban area	Video	96	7days
P17	Male	47	1	Civil servant	25	5,000–10,000	College diploma	Urban area	Video	90	15days
P18	Male	38	2	Bar Manager	1	10,000–15,000	College diploma	Urban area	Video	119	Not returned to work
P19	Male	46	2	Owner	20	>20,000	High school diploma	Urban area	Video	72	15days

### Themes

The study identified thirteen factors influencing the willingness to return to work following interventional procedures among middle-aged and young coronary heart disease patients. These themes are closely aligned with the TDF and the COM-B model; further elaboration is provided in Table 2.

**Table 2:** Mapping of themes to the TDF and the COM-B Model.

Broad components of COM-B Model	Sub-components of COM-B model	TDF	Emerging sub-theme from the transcript
Capability	Physical capability	Skills	1.Physical limitations (-)
	Psychological capability	Knowledge	2.Insufficient knowledge(-)
		Behavioral Regulation	3.Health-oriented behavioral modification (+)
Opportunity	Physical opportunity	Environmental Context and Resources	4.Manageable workloads (+)
	Social opportunity	Social Influences	5.Strong social support networks (+)
		Social Influences	6.Lack of employment opportunities (-)
		Social Influences	7.Descriptive norms (+-)
Motivation	Reflective motivation	Social/Professional Role & Identity	8.Family/professional responsibilities (+)
		Optimism	9.Optimism regarding RTW prospects (+)
		Beliefs about Consequences	10.Belief in the positive outcomes associated with RTW (+)
		Goals	11.Objective of securing economic income (+)
	Automatic motivation	Social/Professional Role & Identity	12.Habitual behaviors influenced by professional identity (+)
		Emotion	13.Emotional responses (+-)

### Capabilities

#### Physical capability

Physical functional limitations represent the most significant obstructive factors impacting patients’ physical capabilities. Some patients continue to experience mild angina symptoms post-surgery. Additionally, participants reported insufficient energy levels and a tendency to feel drowsy and fatigued, which adversely affected their work performance.

P14: “I asked for leave and didn’t go. Because I still keep feeling unwell. Sometimes I feel a bit… uncomfortable in my chest… like stuffy. And sometimes my throat feels a bit tight. Then, after taking the medicine, my stomach often gets a burning sensation. Anyway, I just can’t go to work.”

P16: “There is no way your body can recover in a week, absolutely not. Back then, I felt like I didn’t even have the strength to walk. Then, after going back to work for just one day, I was so weak. It was like having a cold, just sleeping drowsily all the time. Besides, after getting the stent, there were still some uncomfortable reactions.”

#### Psychological capability

Regarding psychological capability, participants demonstrated limited knowledge about physical activity restrictions when returning to work. Additionally, patients expressed uncertainties regarding both their post-PCI prognosis and stent performance. Notably, PCI patients showed positive engagement in health management, demonstrating behavior modification and efforts to balance work demands with health maintenance.

P8: “I was worried about whether heavy work would be affected after getting this stent put in.”

P14: “I was diagnosed with coronary heart disease and angina pectoris. After the stent is implanted, will my coronary heart disease and angina pectoris be cured? Will it not affect me in doing anything?”

P16: “Health is definitely the most important thing, but work is also a matter within the 8-hour workday. If conditions permit, you can arrange the amount or intensity of your work by yourself, as well as adjust your working hours and duration on your own.”

P1: “Work is one thing, but health is important. Health should still come before work! Only by keeping myself healthy can I work well, so I think I should put exercise first in my spare time.”

### Opportunity

#### Physical opportunity

Our study revealed that patients with pre-surgical low-intensity work demonstrated higher return-to-work success rates. This factor significantly facilitated workforce reintegration, as these patients exhibited confidence in resuming their original job roles without requiring workplace accommodations. Moreover, they reported neither encountering occupational challenges nor experiencing reactivation-related stress during the transition.

P5: “My job is quite easy. It doesn’t have any impact on my health, and I don’t have any worries. So I feel that returning to work doesn’t pose any challenges. The current job suits me well, and there’s basically no pressure.”

#### Social opportunity

The TDF domain related to social opportunities is identified as social influences, which in this study encompassed social support, employment opportunities, and descriptive norms. The sources of social support included the workplace and family. Workplaces facilitated return-to-work through proactive strategies such as workload reduction, avoidance of strenuous tasks, and provision of flexible working hours. Additionally, institutional requirements indirectly supported this transition. Family support significantly enhanced patients’ willingness to RTW, a trend that was particularly pronounced among younger patients. Family members perceived returning to work as an indicator of recovery.

P10: “The organization takes good care of me, and the workload is equivalent to taking care of a sick person.”

P5: “My working hours are relatively flexible. If I have something to do, I just let them know and then take a break when I need to. If I don’t go back to work, I’ll have to take long-term sick leave. It’s a state-owned enterprise, after all. If you don’t come to work, you definitely have to explain something to them, right?”

P19: “My family supports me to go back to work because after all I’m only 46 years old.”

P3: “My family also hopes that I can get back to work normally soon, so that everyone can breathe a sigh of relief.”

Regarding employment opportunities, some patients lacked stable workplaces, and their job prospects were subject to market fluctuations. In this study, the limited employment opportunities reported by certain participants significantly diminished their likelihood of returning to work. Employment discrimination also emerged as a critical barrier. Patients without stable employment contracts encountered challenges in post-surgical job hunting. Employers exhibited reluctance to hire individuals with coronary heart disease due to concerns about potential medical emergencies occurring during work hours and the subsequent liability for medical expenses. As a result, patients often felt compelled to conceal their medical conditions during job searches.​.

P9: “There are very few jobs for bridge repair, and there are also few construction projects. Last year was quite good, but this year there are very few projects. So, if we go by last year, there would be a 100% possibility of finding work, but this year it’s only 50% to 60%. When you’re over 60, no one will hire you for work, and you can’t buy endowment insurance or personal safety insurance. If the employer knows you have so many illnesses, they definitely won’t want you.”

P15: “It’s hard to find a job when you’re not allowed to do heavy work. Employers are afraid of taking risks, so they won’t hire you. But patients usually hide their conditions.”

Descriptive norms represent a significant aspect of social influences. Firstly, the peer recovery modeling effect has been shown to enhance patients’ confidence in resuming their normal work and life activities. Patients frequently take the initiative to inquire about the physical recovery experiences of family members, friends, or colleagues who have undergone similar surgical procedures. When they learn that their peers are able to return to regular work, they exhibit a greater willingness to engage in return-to-work efforts, thereby exemplifying the role model effect. Additionally, some patients enter a “semi-retired” state prior to reaching official retirement age. This practice is often tacitly accepted by their workplaces, allowing them the option not to engage in full-time employment.

P13: “Colleagues and friends of my age around me have also had stents placed, and they are all working. I think as long as it’s not heavy physical work or a job with extremely high pressure, I can still work normally.”

P6: “I think having a stent placed doesn’t have a big impact on life. Basically, my body is still okay. For example, some farmers who had stents placed more than 20 years ago are still doing well. They still work and go to the fields to work. I don’t think stents will have a big impact on life.”

P14: “Going back to work is not very important because I am retiring in 4 years and it doesn’t matter whether I go back or not.”

P15: “Going to work doesn’t matter. If the workplace doesn’t ask me to go, I won’t go. I’ll reach retirement age soon. As long as I get paid, it doesn’t matter whether I work or not. Now the workplace is full of young people. When you get to the workplace, what do you think they’ll ask you to do? It’s like this in all county towns.”

### Motivation

#### Reflective motivation

Reflective motivation emerged as a significant theme in this study, with participants indicating that family and professional responsibilities, optimism, beliefs, and economic considerations influenced their willingness to RTW. The participants were predominantly middle-aged men and young adults. As primary breadwinners for their families, their sense of responsibility to support their households served as a strong motivator for returning to work. In addition to familial obligations, some patients pursued an early RTW due to professional commitments.

P8: “I go to work because of family responsibilities, basically all because of family responsibilities. I have a son and a daughter, and they’re both at school. It’s definitely because of family responsibilities, right?”

P1: “Work is definitely important, and I really should go to work on time. I’m about to retire, I should stand my last post.”

P16: “It’s almost Chinese New Year, and we’re an engineering company. Everyone has their own tasks. I was in the hospital for a week, and I still had a lot of things to do, so I only rested for one day before coming back to work.”

Optimism regarding the prospect of returning to work served as a significant facilitator in this context. Participants demonstrated confidence in contemporary medical practices, believing that their reintegration into the workforce would not jeopardize their physical health. Furthermore, some individuals refrained from self-identifying as “patients,” instead viewing themselves as ordinary members of society. This optimistic outlook played a crucial role in motivating their RTW. The findings of this study indicate that physicians’ recommendations concerning the resumption of work bolstered patients’ confidence levels. Participants exhibited unwavering trust in medical professionals and held a strong conviction that they could resume normal occupational and daily activities if advised to do so. Additionally, they complied with doctors’ guidance regarding workload management.

P19: “I don’t feel worried because science is very advanced now. As long as you follow the doctor’s advice, take medicine on time, and have a regular schedule, there will be no problem going back to work.”

P18: “I first let it go in my heart, not thinking, ‘Oh, I’m someone who has had a stent placed.’”.

P10: “Whether to return to work or not should depend on the doctor’s advice. From the patient’s perspective, I think there’s no reason not to follow what the doctor says.”

P12: “Just as the doctor said, there shouldn’t be much of a problem after the operation, and you’ll be just like a healthy person. Life has to go on, and you can’t do without working.”

P2: “The doctor told me not to do work that makes me overly tired, and not to do work that is particularly strenuous and harmful to the body.”

Beliefs that returning to work lead to positive outcomes significantly enhance patients’ willingness to re-enter the workforce. First, several participants indicated that employment positively impacts their physical health. Furthermore, in contrast to remaining at home, patients expressed a desire to restore a sense of normalcy in their lives through engagement in work.

P13: “For me, I think taking part in work is good for physical recovery. Because you have coronary heart disease, you are afraid of blood vessels getting blocked again. I feel that going to work keeps me in a better mood, and with proper exercise, it won’t lead to more severe blockage or re-blockage.”

P7: “There’s nothing to do at home if I don’t go to work, and going to work can make my life more regular.”

Patients explicitly indicated that their primary motivation for work was to generate income. Notably, participants from rural areas highlighted a significant distinction in this context. Unlike their urban counterparts, who have access to pension schemes, rural patients lacked such alternatives. Consequently, they were compelled to secure their income through labor.

P10: “Going back to work brings income. It’s about making money, I tell you.”

P9: “Rural people earn money for each day they work. They are not like urban people who have jobs and pensions. Rural people don’t have pensions. As long as they are capable of working, they have to keep working. When they can’t work anymore, they stop.”

#### Automatic motivation

Automatic motivation plays a pivotal role in participants’ willingness to RTW. It encompasses habitual behaviors associated with professional identity and emotional engagement. Many patients perceived themselves as still young and had not contemplated the possibility of not returning to work. They hold the belief that they must continue working until retirement age. Furthermore, patients often experience feelings of boredom while at home, prompting them to RTW in order to occupy their time. They find it intolerable to lead a life devoid of activity.

P16: “I don’t have too many thoughts about coming back to work. Life has to go on! I’m still young, so what would I do if I don’t go to work? I haven’t thought that much. I still need to go to work.”

P2: “What else would you do at this age if you don’t work! If you’re not yet 60, staying at home would be boring. There’s no need to think about it. It’s not like I have to earn a lot of money. It’s about finding something to do for myself. That’s just how life is. You work as long as you’re alive.”

P8: “I’ve always been someone who can’t stay idle. I couldn’t stay idle even when I was in the hospital. I stayed at home for two days, and then I couldn’t stand being idle anymore, so I came to work.”

Emotion plays a complex role in the decision to return to work. Patients expressed enjoyment in interacting with their colleagues, and this social engagement served as a motivating factor for their RTW. They perceived the heart as a vital organ, and any issues related to it appeared to pose significant threats to their lives. Such anxiety influenced both their willingness to RTW and their emotions. Furthermore, some individuals found struggled with accepting that they had CHD at such a young age. Concerns about potential medical emergencies occurring while at work further exacerbated these feelings. Additionally, some patients experienced guilt due to their poor physical condition necessitating care from family members. Collectively, these factors contributed to an anxious state regarding the decision of whether or not to RTW.​.

P13: “There are more people in the workplace, which makes one feel better. Staying at home, on the other hand, can easily lead to depression.”

P16: “I was in a bad mood those days when I was just diagnosed. Why do people get sick… I even got an old person’s illness. I think many people may not go to work because they consider the heart as the engine of the human body, and if the engine stops, the person dies. Anyway, I’m still very worried.”

P14: “I feel anxious. What if my body suddenly has problems? What if I scare my colleagues and students? My husband cooks for me and washes my clothes every day, but I feel sorry for him… Other people’s wives are all in good health. It’s just that he married a wife who is always in poor health (crying).”

## Discussion

This study identified physical function limitations as the primary factor influencing patients’ ability to return to work, aligning with findings from previous research^19,43,44^. Patients in poor physical condition may opt for early retirement or may not resume work at all^45,46^, potentially due to compromised cardiac function. The left ventricular performance of individuals with coronary heart disease significantly impacts their rate of work resumption and overall work efficiency^19^. Another plausible explanation is that patients frequently experience fatigue during the weeks following discharge, a time when they often lack the energy necessary to engage in practical matters such as employment^44^. Therefore, it is imperative for healthcare providers to prioritize interventions aimed at improving cardiac function and alleviating fatigue in order to enhance patients’ willingness and capacity to return to work.

Young and middle-aged patients with CHD demonstrate a low level of health literacy following interventional surgery, particularly concerning information acquisition^1^. In the current study, physicians encouraged patients to return to their normal work and daily activities; however, they did not provide further guidance regarding work restrictions related to the disease. This observation is consistent with findings from other studies^43,47^. It is essential for doctors to clarify patients’ physical limitations based on their individual health status and occupational demands. Such clarification is crucial for facilitating patients’ RTW efforts. By doing so, it can enhance their post-illness work capacity and increase RTW rates^48^. Furthermore, this study identified a significant lack of disease-related knowledge among patients, which corroborates results from previous research^1^. Patients’ uncertainties regarding disease prognosis and stent performance may lead them to be overly concerned about their physical condition, underestimate their work capabilities, and consequently diminish their willingness to RTW or fully engage in job responsibilities. Therefore, it is imperative that healthcare providers promptly deliver relevant educational resources pertaining to these issues^49^. According to the COM-B theory, behavioral regulation constitutes a vital aspect of psychological capability. The present study revealed that postoperative patients modified detrimental lifestyle habits while striving for a balance between professional obligations and health maintenance—findings that align with prior research outcomes^44^. Notably, individuals who adopt positive health behaviors exhibit higher rates of returning to work compared to those who do not engage in such practices^44^.

The present study found that patients in less demanding jobs were more likely to RTW, which aligns with the findings of previous research^43,50^. Work-related stress may contribute to an increased rate of disability retirements among patients^51^. Conversely, individuals engaged in low-intensity occupations tend to experience higher job satisfaction, which subsequently influences the work resumption rates of CHD patients^19^.

The present study indicated that social support from the workplace or family members facilitated patients’ RTW, aligning with findings from previous research^52–54^. When workplaces reduce the workload for patients and offer flexible working hours, the likelihood of these individuals returning to work increases^49,51,53^. Family members play a crucial role in sharing the stress associated with illness alongside the patient^44^. Such familial support encourages patients to adopt healthier behaviors and aids them in resuming normal life by enhancing their self-efficacy and treatment adherence^1,55^. Furthermore, this study highlighted that young and middle-aged patients with CHD often encounter employment discrimination, which was consistent with findings related to other chronic diseases^56^. The report indicates that approximately 12% of individuals suffering from chronic conditions experience stigma^56^. Strategies aimed at mitigating employment discrimination against these patients are available. One approach involves improving cardiac function; another entails providing health education for employers. This educational initiative helps employers develop an accurate understanding of CHD and its impact on work performance.

Additionally, our study revealed that descriptive norms—such as peer recovery modeling effects and pre-retirement conformity effects—significantly influenced patients’ decisions regarding their RTW. The successful experiences of peers who have resumed work can alleviate concerns among patients about their own health status and adaptability in a professional setting. The pre-retirement conformity effect arises when patients identify socially with the behaviors exhibited by their peer groups. As they internalize ceasing work upon nearing retirement as a “reasonable stage-specific choice,” they may align their actions with those of others in their group, thereby diminishing their willingness to RTW.

This study found that family responsibilities emerged was a critical factor influencing patients’ willingness to RTW. For many patients, resuming work is an instinctive action driven by their obligation to support their children, which aligns with research on the return-to-work behavior of AMI^43^. Furthermore, this study identified work responsibility as another significant motivator for returning to work. For some cardiovascular disease patients, employment constitutes a central aspect of their lives^49^. Patients may perceive their responsibilities as irreplaceable. This “responsibility anxiety” prompts them to expedite recovery and RTW earlier. The present study indicated that some patients maintained an optimistic attitude toward disease recovery and RTW, harboring positive expectations for their future social functioning. This finding is consistent with Mari’s research^44^. Patients’ understanding of their condition and ability to return to work largely depend on guidance from physicians; they expect healthcare providers to offer positive and effective advice regarding their future professional and personal lives^19,43^. In this study, doctors encouraged patients to engage in normal work and life activities, which contrasted sharply with findings from Turkish scholars^43^ who reported different perspectives. Some physicians advised patients—without fully understanding their occupational circumstances—to prioritize physical health above all else, warning that neglect could lead to severe consequences such as death. Such statements can contribute significantly to feelings of depression and despair among patients, ultimately hindering their ability or motivation to return to the workforce. Therefore, it is imperative that healthcare professionals provide timely positive feedback tailored appropriately for each patient’s situation. The study revealed that patients’ perception of the health benefits associated with returning to work motivated them to resume their professional activities. This perception not only reinforces patients’ understanding of the significance of re-entering the workforce but also enhances their intrinsic motivation to proactively seek employment opportunities. Some patients expressed that returning to work allowed them to regain a sense of normalcy in their lives. Engaging in daily routines and habits can assist patients in alleviating stress and anxiety, redirecting their focus from illnesses and symptoms toward more positive aspects of life^44^. The connection between returning to work and the necessity for financial income has been substantiated by numerous studies^43,47^. Patients experiencing physical discomfort often choose to return to work primarily due to concerns about losing their source of income^57^.

Habitual behaviors related to professional identity serve as a primary factor influencing patients’ automatic motivation to RTW. The present study indicated that, for certain patients, returning to work was a habitual behavior closely linked with their professional identity. These individuals view continuous employment as an essential aspect of their daily activities. By sustaining such behavioral inertia, they mitigate the psychological discomfort associated with role transitions, thereby facilitating their RTW. Psychosocial factors significantly impact the likelihood of returning to work among patients with cardiovascular diseases^58^. This study found that post-surgery, many patients experienced negative emotions—including anxiety, depression, fear of death and recurrence, and self-blame—which adversely affected their willingness to re-enter the workforce. These findings align with those reported in previous studies^43,58,59^. In recent years, multi-vessel lesions have increasingly been observed in younger patients^2^. During hospitalization, these individuals may experience depressive symptoms^60^, and even one year later may still grapple with feelings of depression and despair^44^. Additionally, some patients express anxiety regarding potential disease recurrence^61^. Such anxiety may stem from a lack of clear guidelines concerning the rehabilitation process; many do not know when they can safely resume normal work and life activities^44^. This uncertainty contributes to psychological distress and pressure on patients while also impacting their willingness to return to work^59^.

## Conclusion

This study identified the barriers and facilitators to RTW among young and middle-aged patients following PCI, utilizing the COM-B framework. Physical limitations, a lack of knowledge, and insufficient employment opportunities impede patients’ ability to return to work. Conversely, low workload, social support, personal responsibilities, optimism, beliefs, economic factors, and habitual behaviors serve as facilitators for RTW. Additionally, descriptive norms and emotional states can function as both facilitators and barriers. Enhancing patients’ cardiac function alongside providing comprehensive patient education is essential. Strengthening social support networks while fostering optimistic attitudes and beliefs regarding RTW will also contribute positively. Furthermore, addressing negative emotions is crucial in improving patients’ willingness to engage in RTW activities. Overall, the findings of this study carry significant implications for healthcare professionals and policymakers in designing personalized interventions that strengthen patient education initiatives and promote vocational rehabilitation strategies.

### Limitations of the study

Our study has several limitations. First, the majority of participants were male and from urban areas, which may reflect the epidemiological factors associated with CHD. To enhance the robustness of our findings, it is essential to involve more hospitals and recruit a greater number of female and rural patients for interviews. This approach could yield more comprehensive insights into the subject matter. Second, our data primarily reflect experiences during the early recovery phase and may not fully capture the challenges and adjustments throughout the entire, often prolonged, return-to-work process. Future longitudinal studies are warranted to delineate its dynamic trajectory. Additionally, the scope of this research was confined to Xi’an. Consequently, future studies should prioritize multi-center interviews to achieve a broader and more representative understanding of the factors influencing RTW among patients following PCI. Lastly, while this study utilized WeChat video interviews as a methodological tool, there are inherent limitations associated with this approach. It is anticipated that face-to-face interviews in future research will facilitate the collection of more meaningful data.

## Data Availability

The data that support the findings of this study are available from the corresponding author, YZ, upon reasonable request.

## Appendix 1: Interview schedule based on TDF

**Table:**

TDF	Definition	Interview guide
Knowledge	An awareness of the existence of something	Do you think returning to work is important? Why do you think so?
Skills	An ability or proficiency acquired through practice	Which skills or capabilities do you feel need enhancement in order to fulfill the demands of returning to work successfully?
Social/professional role and identity	A coherent set of behaviors and displayed personal qualities of an individual in a social or work setting	In light of your physical condition following surgery, do you believe that adjustments are necessary regarding your job responsibilities? What specific modifications do you think are required? How do you intend to balance professional obligations with health considerations? What type of care and support has your workplace or employer provided you with during this transition?
Beliefs about capabilities	Acceptance of the truth, reality, or validity about an ability, talent, or facility that a person can put to constructive use	How confident are you in managing any difficulties or challenges that may emerge upon re-entering the workforce? Do you feel that you possess adequate decision-making authority and resources concerning your return-to-work plans? What specific resources are included in this assessment?
Optimism	The confidence that things will happen for the best or that desired goals will be attained	How believe it is that you will return to work?
Beliefs about consequences	Acceptance of the truth, reality, or validity about outcomes of a behavior in a given situation	What benefits do you foresee from returning to work? What consequences might arise if you choose not to return? How could this impact your health overall? (Tips: Consider both the potential benefits and drawbacks.)
Reinforcement	Increasing the probability of a response by arranging a dependent relationship, or contingency, between the response and a given stimulus	Which factors do you believe will motivate your return to work? What consequences might arise if you choose not to return? Are you aware of anyone who has been diagnosed with coronary heart disease or who has undergone surgery for other medical conditions? Are they currently employed? Have there been any repercussions associated with not returning to work? Conversely, have those who have returned managed to recover physically and perform effectively in their roles?
Intentions	A conscious decision to perform a behavior or a resolve to act in a certain way	Do you plan to return to work? What circumstances have previously hindered your ability to return or caused interruptions in your employment?
Goals	Mental representations of outcomes or end states that an individual wants to achieve	When do anticipate resuming your duties?
Memory, attention and decision processes	The ability to retain information, focus selectively on aspects of the environment and choose between two or more alternatives	What decisions must be addressed during the process of returning to work? Do you believe that this decision necessitates careful consideration and a weighing of its advantages and disadvantages? (Tips: Is making this decision challenging for you? )
Environmental context and resources	Any circumstance of a person’s situation or environment that discourages or encourages the development of skills and abilities, independence, social competence, and adaptive behavior	Which factors do you believe will motivate your return to work, and which may impede it?
Social influences	Those interpersonal processes that can cause individuals to change their thoughts, feelings, or behaviors	Do your family members, friends, colleagues, and healthcare providers support your decision to return to work? Why? Are you aware of anyone who has been diagnosed with coronary heart disease or who has undergone surgery for other medical conditions? Are they currently employed? Will you consider comparing yourself to them when determining the appropriate time for your own return to work?
Emotion	A complex reaction pattern, involving experiential, behavioral, and physiological elements, by which the individual attempts to deal with a personally significant matter or event	How do you feel about the prospect of returning to work? Will these feelings or emotions affect your willingness to return to work? Do you have any concerns regarding this transition?
Behavioral regulation	Anything aimed at managing or changing objectively observed or measured actions	What measures or strategies do you propose could facilitate the reintegration of patients into the workforce after undergoing interventional surgery?
